# Structural and functional alterations of posterior cingulate cortex subregions in successful cognitive aging

**DOI:** 10.3389/fnagi.2026.1929453

**Published:** 2026-09-01

**Authors:** Jie Wang, Yunfei Li, Tianyuyi Feng, Ruixia Xu, Jiaqi Li, Yanling Zheng, Yang Hu, Xiaohu Zhao

**Affiliations:** 1 The Department of Radiology of the Fifth People’s Hospital of Shanghai, Fudan University, Shanghai, China; 2 Department of Radiology, Sichuan Provincial People’s Hospital, University of Electronic Science and Technology of China, Chengdu, China; 3 Department of Radiology, Deyang People’s Hospital, Deyang, China; 4 Chongqing BLCX Technology Co., Ltd., Chongqing, China

**Keywords:** cortical microstructure, functional connectivity, multimodal neuroimaging, posterior cingulate cortex, successful cognitive aging

## Abstract

Successful cognitive aging (SCA) describes the preservation of cognitive abilities into advanced age, but its neural basis remains incompletely understood. Metabolic and pathological evidence implicates the posterior cingulate cortex (PCC) as a key region supporting cognitive resilience. However, functionally distinct PCC subregions have rarely been examined using complementary structural, microstructural, and functional imaging measures. In this cross-sectional secondary analysis of publicly available Human Connectome Project in Aging data, we identified 29 adults aged at least 80 years who met successful cognitive aging criteria based on episodic memory and executive function. We selected 29 adults with normal aging (NA) group and 29 middle-aged adults (MA) group matched for sex and education, yielding 87 participants. We combined structural MRI, neurite orientation dispersion and density imaging, and resting-state functional MRI to assess multimodal features across three PCC subregions. These features included cortical thickness, Neurite Density Index (NDI), Orientation Dispersion Index (ODI), Free Water Fraction (FWF), and cortex-wide functional connectivity. Both older groups showed lower cortical thickness in the dorsal and ventral PCC than the MA group. Cortical thickness, NDI, and ODI did not differ significantly between successful and normal aging. FWF was numerically lower in the left ventral PCC in SCA group, but this exploratory observation did not reach significance (Tukey-adjusted *P* = 0.059). Compared with the NA group, the SCA group showed significantly weaker connectivity between all PCC subregions and widespread prefrontal cortices (adjusted *P* < 0.05). In the pooled older-adult sample, weaker left ventral PCC–medial prefrontal connectivity correlated with better episodic memory (*r* = −0.430, FDR-adjusted *P* = 0.026), although neither within-group association was significant. These findings suggest that SCA was associated with lower PCC–prefrontal functional connectivity, but not with detectable preservation of PCC cortical thickness. The numerical FWF difference in the left ventral PCC remains exploratory and requires independent replication. These subregion-specific patterns provide a potential framework for understanding heterogeneous cognitive-aging trajectories, but require confirmation in larger longitudinal cohorts.

## Introduction

While cognitive decline is a prevalent hallmark of aging, often progressing to pathological states such as dementia, a subset of older adults maintains cognitive function comparable to that of younger individuals. This phenomenon is defined as Successful Cognitive Aging (SCA) (Depp et al., 2012). Individuals exhibiting SCA possess cognitive abilities and functional independence superior to their age-matched peers, a phenotype essential for preserving quality of life in late adulthood (Krivanek et al., 2021). Given its profound clinical implications, accurately characterizing this elite cohort has become a major research focus. A recent systematic review grouped operational definitions of SCA into generational-comparison, cross-sectional-comparison, and longitudinal-tracking approaches (Yang et al., 2026). Despite the variation in operational criteria across studies, the theoretical consensus identifies a specific resistance to age-related episodic memory decline as its core phenotypic hallmark (Depp et al., 2012; Harrison et al., 2012; Krivanek et al., 2021). Although existing evidence suggests that genetic backgrounds (e.g., APOE and BDNF variants) and lifestyle interventions (e.g., high-intensity aerobic exercise, sustained cognitive engagement, and social participation) play pivotal roles in promoting SCA (Raichlen and Alexander, 2014, 2017), the specific underlying neurobiological mechanisms remain elusive. Consequently, elucidating these mechanisms is of critical scientific and clinical significance. Such insights may inform future research on potential intervention targets and strategies to promote healthy cognitive aging.

Non-invasive magnetic resonance imaging (MRI) has provided a crucial window into the neural substrates of SCA. Structural MRI studies have shown that successful agers exhibit greater cortical preservation than typical older adults, with the most consistent differences involving the anterior cingulate cortex (ACC), hippocampus, posterior cingulate cortex (PCC), and prefrontal regions (Yang et al., 2026). Harrison et al. (Harrison et al., 2012; Pezzoli et al., 2024) further reported that selected ACC regions in successful agers were preserved or even thicker than those in middle-aged controls. Similarly, Sun et al. found greater cortical thickness in key nodes of the default mode network (DMN) and salience network (SN) in successful agers (Sun et al., 2016), while resting-state fMRI studies have reported altered, and in some cases stronger, functional connectivity within these networks (Xu et al., 2023; Zhang et al., 2020). Although structural preservation has been less consistently reported in the posterior cingulate cortex (PCC), accumulating multimodal evidence also implicates posterior cingulate regions in SCA. Longitudinal MRI has revealed slower gray-matter volume loss in the right PCC (Garo-Pascual et al., 2023), proton magnetic resonance spectroscopy has shown higher total N-acetylaspartate concentrations in the PCC (de Godoy et al., 2021), and functional studies have identified altered PCC-related connectivity (Lin et al., 2017) and the PCC as a discriminative node for the SuperAging phenotype (Park et al., 2022). Moreover, PET studies demonstrated higher global cortical glucose metabolism in cognitively successful older adults, with the right isthmus cingulate – a posterior cingulate territory – showing both relatively preserved glucose metabolism and lower amyloid burden (Baran and Lin, 2018). Together, these findings suggest that PCC involvement in SCA may extend beyond macroscopic structural preservation and may differ across biological measures and functional connections. However, it remains unclear whether these alterations are distributed uniformly across the PCC or are specific to particular PCC subregions at the microstructural and functional levels.

To fully unravel the neural mechanisms of SCA, we must move beyond global average metrics and investigate core brain regions that exhibit the highest sensitivity to the aging process. Modern neuroimaging evidence indicates that brain aging is not uniform but follows a pattern of “selective vulnerability (Seeley et al., 2009).” Large-scale functional networks responsible for high-order cognitive integration – particularly the DMN – are often the primary targets for early amyloid deposition, hypometabolism, and cortical atrophy (Buckner et al., 2005). As the anatomical anchor and functional hub of the DMN, the Posterior Cingulate Cortex (PCC) occupies a unique position in the brain’s metabolic landscape: it exhibits the highest resting-state metabolic rate and serves as a critical node for maintaining whole-brain communication efficiency (Leech and Sharp, 2014). This high metabolic demand places the PCC at the center of a “risk-function” trade-off. In pathological aging, such as Alzheimer’s disease (AD), the PCC often acts as the “epicenter” of network collapse (Lee et al., 2020). Given its critical role in pathological trajectories, we posit that focusing on the structural and functional characteristics of the PCC may provide insight into the neural features associated with SCA.

However, the majority of prior studies have treated the PCC as a homogeneous functional entity (Leech et al., 2011; Zhang et al., 2014), a methodological limitation that may mask subregion-specific alterations occurring during aging. Strong evidence supporting PCC heterogeneity comes not only from pathological aging but increasingly from SCA research. In AD, the dorsal and ventral subregion of PCC exhibit distinct functional connectivity patterns and pathological susceptibilities (Mutlu et al., 2016); indeed, debate persists regarding whether AD pathology originates in the memory-related ventral subregion or the executive control-related dorsal subregion (Leech and Sharp, 2014). This heterogeneity strongly suggests that potential neuroprotective mechanisms in SCA are likely subregion-specific. For instance, Baran et al. found that “SuperAgers” exhibited specific low amyloid deposition and high glucose metabolism in the right isthmus cingulate (Baran and Lin, 2018), suggesting that SCA-related imaging characteristics may be regionally specific rather than uniformly distributed across the PCC. From a functional anatomy perspective (Foster et al., 2023; Leech and Sharp, 2014), the PCC can be parcellated into three heterogeneous subregions: the dorsal PCC (dPCC), which primarily supports executive control systems and couples with attention networks; the ventral PCC (vPCC), a core DMN node closely linked to episodic memory; and the retrosplenial cortex (RSC), which plays a critical role in spatial processing and navigation. Therefore, dissecting the differential maintenance mechanisms of these subregions is crucial for understanding how the brain resists specific pathological insults.

Given the significant heterogeneity in cytoarchitecture and functional connectivity among the dPCC, vPCC, and RSC, we hypothesize that the core role of the PCC in SCA is not characterized by uniform, global protection, but rather by “selective protection” of specific critical subregions. This mechanism is likely driven not by a single biological dimension, but by a synergistic interplay across macroscopic, microscopic, and functional levels. Previous research, often limited to a single scale, has struggled to capture this multi-dimensional dynamic balance. To comprehensively deconstruct this mechanism, the current study innovatively employs a multi-modal, multi-scale neuroimaging approach to systematically explore consistent or subregion-specific changes within the three PCC subregions in SCA individuals: (1) At the macroscopic morphological level, we utilized high-resolution T1-weighted imaging to assess cortical thickness as foundational metrics of neuroprotection; (2) At the microstructural level, we employed the advanced Neurite Orientation Dispersion and Density Imaging (NODDI) model to quantify neurite microstructure, specifically deriving the neurite density index (NDI), the orientation dispersion index (ODI), and the free water fraction (FWF); (3) At the functional network level, we applied resting-state fMRI (rs-fMRI) to map subregion-specific functional connectivity profiles. This multimodal, multiscale design allows complementary assessment of PCC subregions without treating the PCC as a homogeneous unit. Our core hypothesis is that SCA is associated with heterogeneous, subregion-specific differences across macroscopic structure, microstructural organization, and functional networks. This study aims to characterize the subregion-specific neuroimaging features associated with SCA and provide a more refined framework for understanding heterogeneity in cognitive aging.

## Materials and methods

### Participants and cognitive assessments

Data were obtained from the Human Connectome Project in Aging (HCP-Aging) cohort. During recruitment, the HCP-Aging study excluded individuals with known major neurological disorders and applied age-specific cognitive screening criteria. According to the three-type framework, operational definitions of successful cognitive aging (SCA) generally fall into three methodological paradigms: (1) single-domain extreme-value criteria focusing on exceptional episodic memory (Harrison et al., 2012); (2) multidomain composite scores derived from comprehensive neuropsychological batteries (Krivanek et al., 2021); and (3) longitudinal tracking to confirm the absence of cognitive decline over time (Depp et al., 2012). While comprehensive batteries and long-term follow-ups provide robust theoretical insights, they impose heavy testing burdens on older adults and are logistically prohibitive in routine clinical settings. Recognizing that specific resistance to age-related episodic memory decline is the core phenotypic hallmark of SCA, the classic conceptualization requires a dual-domain evaluation pairing this exceptional memory with preserved non-memory cognition (executive function) (Harrison et al., 2012; Keenan et al., 2024). Therefore, we selected the Rey Auditory Verbal Learning Test (RAVLT) and the Trail Making Test Part B (TMT-B) from the available dataset, as they represent the two pivotal indicators that best reflect the core characteristics of SCA. This targeted combination leverages measures that are highly feasible and easily deployable in routine clinical practice while retaining the core cognitive dimensions commonly used to define SCA.

Based on previous study (Cook et al., 2017), the SCA group was defined as participants aged 80 years or older who met the reference criteria for episodic memory and executive function. The episodic memory criterion was a RAVLT delayed-recall score of ≥9, the nearest whole-number cutoff to the mean score of 9.37 in the eligible 50–65-year-old reference cohort before matching. The executive-function criterion was a TMT-B completion time of ≤117.07 s, corresponding to the mean of the eligible age-matched older reference cohort before matching. The NA group comprised participants aged 80 years or older with RAVLT delayed-recall scores below the middle-aged reference cutoff (<9). The MA group comprised participants aged 50–65 years who met the corresponding age-specific criteria for episodic memory and executive function. Before matching, 29 SCA, 63 NA, and 164 MA participants met the eligibility criteria. Using the SCA group as the reference, we selected 29 participants from each of the NA and MA candidate pools to obtain comparable distributions of sex and years of education across groups. When multiple candidates met the matching criteria, participants were selected according to their order in the original HCP-Aging data file. This procedure yielded a final balanced sample of 29 participants per group. The complete participant-selection procedure is shown in Supplementary Figure 1.

### MRI dataset

MRI data were acquired across four HCP-Aging sites using a common Siemens 3T Prisma platform, the same software version, and an electronically distributed acquisition protocol. Standardized operating procedures and centralized quality-control procedures were implemented to minimize intersite variability. Initial HCP-Aging quality-control analyses indicated that acquisition site explained less than 10% of the variance in selected resting-state fMRI quality-control measures, including temporal signal-to-noise ratio, frame-to-frame motion, and image smoothness (Bookheimer et al., 2019; Harms et al., 2018). For each participant, one T1-weighted image, one T2-weighted image, 4 runs of resting-state fMRI images, and 4 runs of diffusion-weighted images were acquired. For T1 and T2 images, a spatial resolution of 0.8 mm was used; For rs-fMRI, data were acquired at 2 mm spatial resolution, with a TR of 0.8 s and 488 volumes per run. Two runs used anterior-to-posterior (AP) encoding directions, while the other two used posterior-to-anterior (PA) encoding directions. Additionally, spin-echo field maps with reversed phase-encoding directions were acquired to correct geometric distortions. For diffusion images, four runs were collected at 1.5 mm spatial resolution. Two runs employed 98 diffusion-weighting directions (AP and PA encoding), while the other two employed 99 directions (AP and PA encoding). Each run included a dual-shell acquisition with b-values of 1500 and 3000 s/mm^2^, and 7 b = 0 s/mm^2^ volumes. Detailed sequence-specific MRI acquisition parameters are provided in the Supplementary Methods and have been reported previously in the original HCP-Aging protocol publications.

### MRI preprocessing and quality control

MRI data were preprocessed using HCP Pipelines (v5.0.0) (Glasser et al., 2013). The HCP Pipelines leverage several software packages, including FreeSurfer (v6.0.0) (Dale et al., 1999), FSL (v6.0.7.16) (Jenkinson et al., 2002), Connectome Workbench (v2.0.1) (Marcus et al., 2011), MSM (v3) (Robinson et al., 2014), to implement specific preprocessing steps. For T1- and T2-weighted images, the preprocessing steps mainly included: registration between T1 and T2, registration between T1 and the MNI152 template, surface reconstruction and standardization, and extraction of morphological features. For resting-state fMRI images, the preprocessing steps mainly included: motion correction, distortion correction, registration between fMRI and T1-weighted images (Greve and Fischl, 2009), using multi-run FIX (Salimi-Khorshidi et al., 2014) to mitigate motion-related and respiratory noises, and refined surface alignment via the MSMAll approach. The fMRI data in the standard surface space were subsequently used for functional connectivity analysis. For diffusion images, preprocessing steps mainly included: b0 intensity normalization, distortion correction (Andersson et al., 2003), eddy and motion correction (Andersson and Sotiropoulos, 2016), registration between b0 and T1-weighted images, and transformation into T1-weighted space for further microstructural analysis.

For the quality control of T1/T2-weighted preprocessing, we visually checked the accuracy of surface reconstruction and T1-MNI152 registration. We also extracted the Euler number from the recon-all result, which could be used as a quantitative metric of raw data quality (Rosen et al., 2018). The Euler number would be treated as a covariate in the later statistical analysis to account for the influence of image quality on the morphological features. For the quality control of resting-state fMRI preprocessing, we visually checked the accuracy of registration between fMRI and T1 data. We calculated the mean Jenkinson’s frame-wise displacement (MeanFD) to quantify the overall head motion and ensured the MeanFD of each fMRI run to be lower than 0.3 mm. For the quality control of diffusion preprocessing, we visually checked the accuracy of registration between diffusion and T1 images. We calculated the mean contrast-to-noise ratio (MeanCNR) based on the eddy correction result to represent the overall data quality (Bastiani et al., 2019). The MeanFD and MeanCNR would be considered as covariates in the corresponding statistical analyses to control the influence of image quality on the functional and microstructural features.

### Delineation of posterior cingulate subregions

**FIGURE 1:**
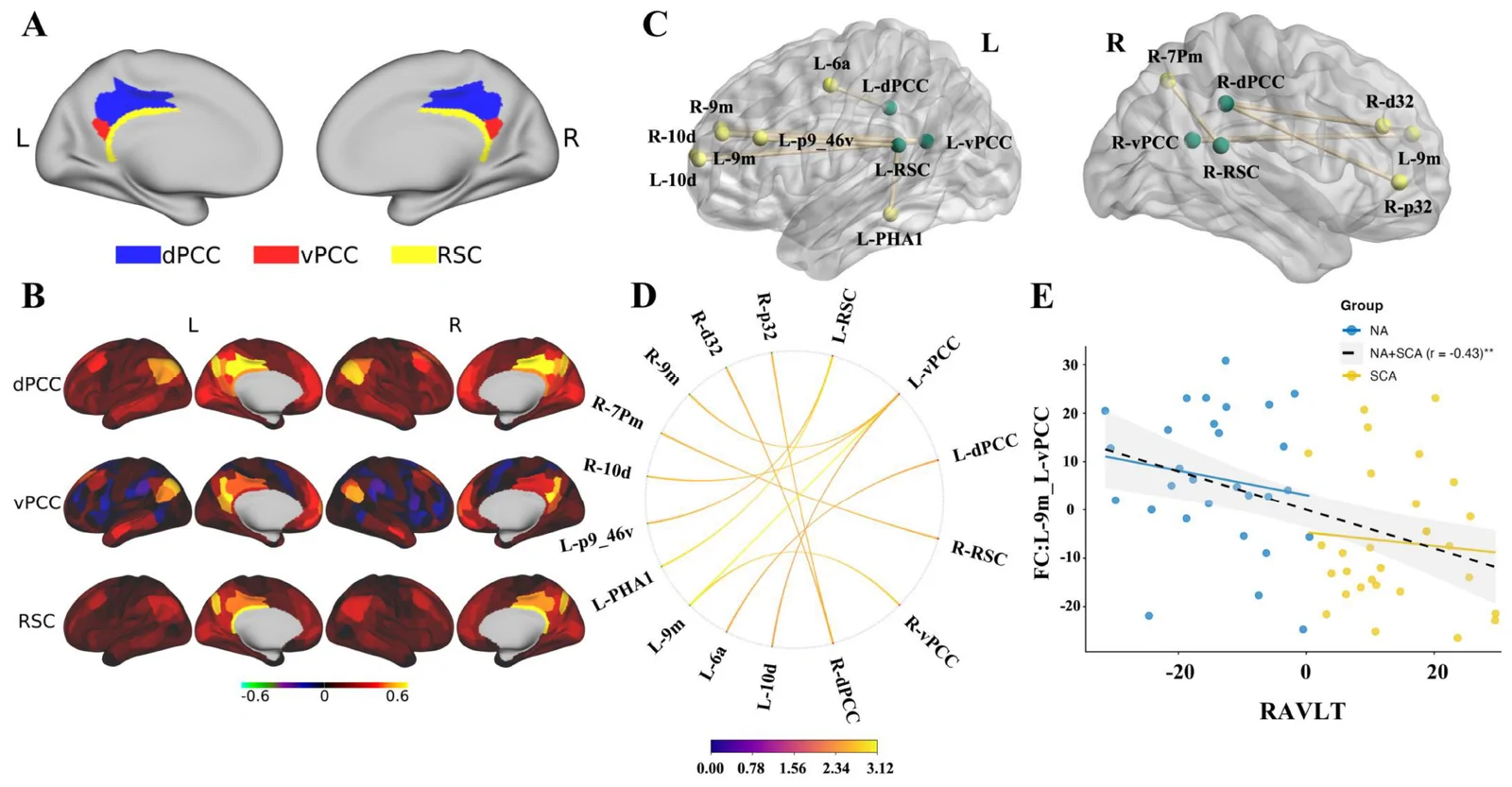
Subregion-specific functional connectivity alterations in successful cognitive aging and their associations with episodic memory. **(A)** Parcellation of the posterior cingulate cortex (PCC) based on the HCP-MMP atlas into the dorsal PCC (dPCC, blue), ventral PCC (vPCC, red), and retrosplenial cortex (RSC, yellow). **(B)** Seed-based resting-state functional connectivity (FC) profiles of the dPCC, vPCC, and RSC across both hemispheres. The color scale represents Pearson’s correlation coefficients. **(C)** Three-dimensional cortical visualization of FC edges showing lower connectivity in the SCA group than in the NA group. The labels L and R denote edge sets involving left- and right-hemisphere PCC seed nodes, respectively, rather than the hemispheric location of every connected target region. Target regions may therefore be located in either hemisphere. The identified edges predominantly connected PCC subregions with prefrontal regions, including the medial prefrontal cortex (e.g., areas 9m and 10d) and anterior cingulate cortex (e.g., areas d32 and p32). **(D)** Circular chord diagram summarizing the region-to-region FC differences between the SCA and NA groups. Edge colors represent the T statistics for the NA > SCA contrast. Edges shown in panels C and D satisfied an FDR-adjusted omnibus group effect followed by a significant SCA–NA post hoc comparison (Tukey-adjusted *P <* 0.05). **(E)** Association between left vPCC–left area 9m connectivity and RAVLT delayed-recall performance. Partial Spearman correlation analysis, adjusted for age, sex, years of education, and mean framewise displacement, showed a significant negative association in the combined older-adult sample (SCA and NA, *n* = 58; *r* = −0.430, raw *P* = 0.0012, FDR-adjusted *P* = 0.026). The association was not significant within the SCA group (*n* = 29; *r* = −0.085, *P* = 0.687) or the NA group (*n* = 29; *r* = −0.164, *P* = 0.433). The black dashed line represents the pooled fit, whereas the colored solid lines represent group-specific fits. The gray band denotes the 95% confidence interval for the pooled fit. Because the within-group associations were not significant, the pooled association may partly reflect between-group differences and should be interpreted accordingly.

We obtained the HCP-MMP parcellation atlas (Glasser et al., 2016) from the BALSA database^1^. This atlas parcellates the cerebral cortex into 360 distinct areas. Based on previous studies (Foster et al., 2023; Vogt et al., 2006), we selected the RSC area to represent the retrosplenial cortex, the v23ab area to represent the ventral PCC (vPCC), and combined the d23ab, 31pv, 31pd, 31a, 23d, and 23c areas to represent the dorsal PCC (dPCC). The spatial distributions of the bilateral dPCC, vPCC, and RSC regions are shown in Figure 1A.

### Multi-modal feature extraction

For T1/T2-weighted data, the average cortical thickness for each PCC subregion was calculated.

For resting-state fMRI data, we extracted the mean time series from each PCC subregion as well as from all other cortical areas defined by the HCP-MMP atlas. Pearson’s correlation coefficients were then computed between each PCC subregion and every other cortical region, as well as between the PCC subregions themselves. The resulting correlation values were subsequently Fisher’s Z-transformed to serve as the measure of functional connectivity between cortical regions.

For diffusion data, we fitted the NODDI model (Zhang et al., 2012) in each voxel using the AMICO package (v2.0.3) (Daducci et al., 2015) to derive three microstructural features: neurite density index (NDI), orientation dispersion index (ODI), and free water fraction (FWF). To optimize the model fit in gray matter, the intrinsic parallel diffusivity was set to 1.1 × 10^–3^ mm^2^/s (Fukutomi et al., 2018). The PCC subregions were then transformed from the standard surface space to native volumetric space. NDI and ODI characterize properties of the tissue compartment, and their regional estimates can be biased by cerebrospinal-fluid partial-volume contamination in cortical voxels. We therefore calculated tissue-weighted means for NDI and ODI using the estimated tissue fraction, thereby reducing the contribution of voxels with greater isotropic or free-water contamination^44^. In contrast, FWF represents the isotropic free-water-like compartment itself. Applying the same tissue weighting to FWF would down-weight the quantity being measured and alter its interpretation as the regional free-water fraction; therefore, FWF was summarized using the conventional arithmetic mean (Parker et al., 2021).

### Statistical analysis

For cognitive assessment data, a one-way ANCOVA was performed to assess group differences, with sex and years of education included as covariates. The same ANCOVA framework was applied to multimodal MRI brain features. Specifically, for cortical thickness, sex, years of education, and Euler number were modeled as covariates; for functional connectivity, sex, years of education, and meanFD were included; for the NDI, ODI, and FWF metrics, sex, years of education, and meanCNR were incorporated.

In each ANCOVA model, we first examined the significance of the group main effect. Multiple comparison correction was performed using the Benjamini–Hochberg false discovery rate (FDR) procedure. When a significant group main effect was observed, post-hoc analyses were conducted using Turkey’s test, which also controlled for multiple comparisons.

**FIGURE 2:**
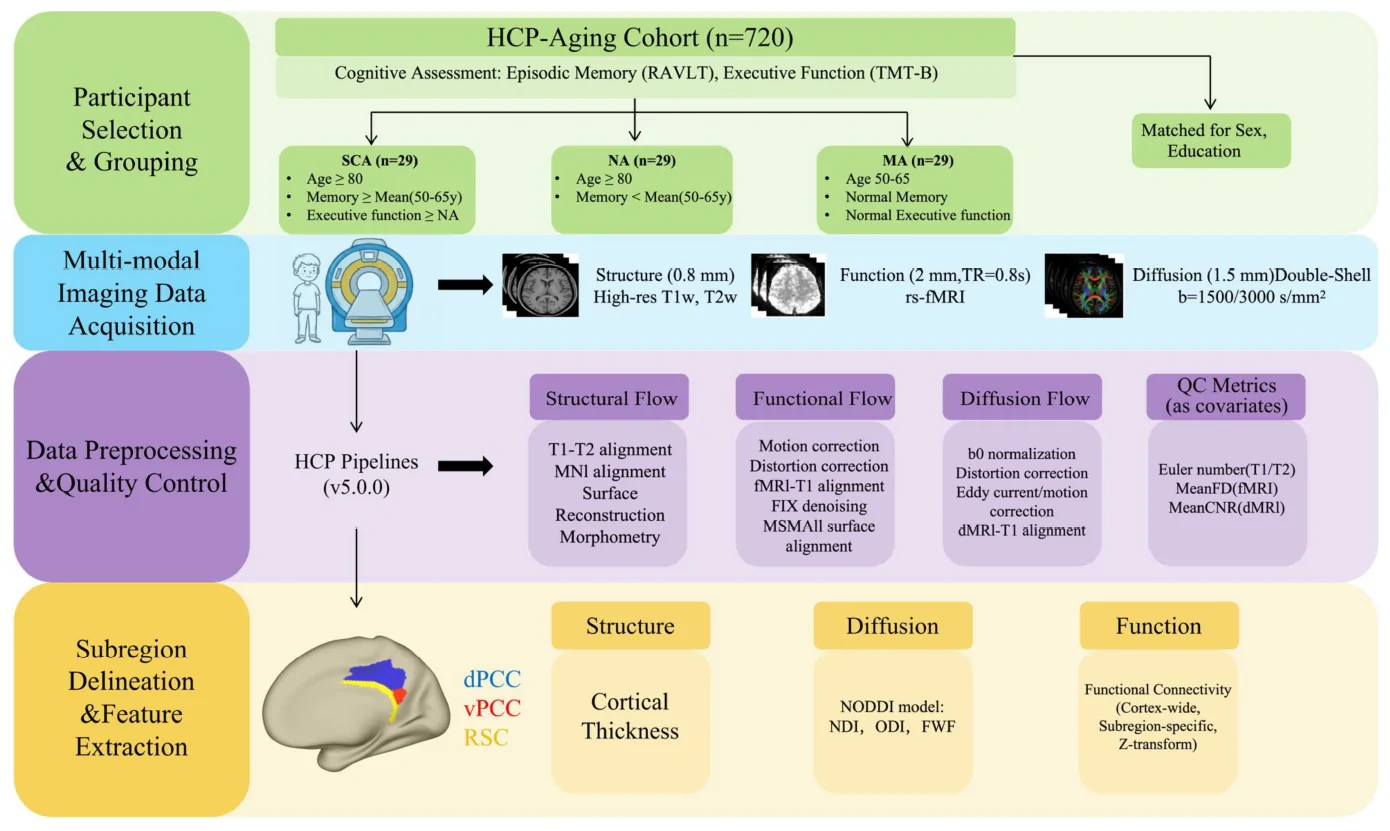
Flowchart of participant grouping and multimodal neuroimaging analysis based on the HCP-Aging cohort. The analytical pipeline comprised four main steps. (1) Participant grouping: Participants were classified as successful cognitive aging (SCA), normal aging (NA), or middle-aged adults (MA) based on RAVLT and TMT-B performance. NA and MA participants were matched to the SCA group for sex and years of education, yielding 29 participants per group. (2) Data acquisition: High-resolution T1- and T2-weighted structural MRI, resting-state functional MRI (rs-fMRI), and double-shell diffusion MRI (dMRI) data were acquired. (3) Preprocessing and quality control: Multimodal MRI data were processed using the HCP Pipelines (v5.0.0). Euler number, mean framewise displacement (MeanFD), and mean contrast-to-noise ratio (MeanCNR) were used as modality-specific image-quality covariates. (4) Feature extraction: Cortical thickness, NODDI-derived microstructural parameters (NDI, ODI, and FWF), and cortex-wide functional connectivity were extracted for the dPCC, vPCC, and RSC. SCA, successful cognitive aging; NA, normal aging; MA, middle-aged adults; RAVLT, Rey Auditory Verbal Learning Test; TMT-B, Trail Making Test Part B; PCC, posterior cingulate cortex; dPCC, dorsal posterior cingulate cortex; vPCC, ventral posterior cingulate cortex; RSC, retrosplenial cortex; NODDI, neurite orientation dispersion and density imaging; NDI, neurite density index; ODI, orientation dispersion index; FWF, free water fraction; MeanFD, mean framewise displacement; MeanCNR, mean contrast-to-noise ratio.

For imaging features that differed significantly between the SCA and NA groups, partial Spearman correlations with cognitive measures were calculated separately within each group and additionally in the combined older-adult sample to mitigate range restriction (Bland and Altman, 2011), with age, sex, years of education, and image quality metrics included as covariates. The FDR approach was also applied to control for multiple comparisons. All statistical analyses were conducted using R (v4.4.2). The specific experimental procedure can be found in Figure 2.

Although the HCP-A data were acquired at four sites, we did not adjust the site effect in our primary analysis, because the site variability was minimized during data acquisition and no perfect algorithm exists for adjusting site effect. To ensure the current results were not driven by site effect, we first compared the acquisition-site distributions across the MA, NA, and SCA groups using Fisher’s exact test because of the relatively small cell counts in the site-by-group contingency table. We then performed a sensitivity analysis using ComBat (Fortin et al., 2018), implemented in the combat.enigma (Radua et al., 2020) package (v1.1.1), with acquisition site specified as the batch variable. The same ANCOVA, FDR-correction, and Turkey *post hoc* procedures were subsequently repeated using the ComBat-harmonized measures.

## Results

### Demographic and cognitive characteristics

As shown in Table 1, the SCA and NA groups did not differ significantly in age (85.27 vs. 84.63 years), sex distribution, or years of education. The MA group was matched to the SCA group for sex and years of education but was younger by definition. As expected from the operational classification criteria, RAVLT delayed-recall scores were higher in the SCA group than in the NA group (10.44 ± 1.38 vs. 5.79 ± 1.54; Tukey-adjusted *P* < 0.001) and did not differ significantly between the SCA and MA groups (10.44 ± 1.38 vs. 10.41 ± 1.55; Tukey-adjusted *P* = 0.84). TMT-B completion time was shorter in the SCA group than in the NA group (78.80 vs. 102.21 s; Tukey-adjusted *P* < 0.05) but longer than in the MA group (52.04 s; Tukey-adjusted *P* = 0.002). Because RAVLT and TMT-B performance contributed to the operational group definitions, these differences were considered expected consequences of the classification criteria rather than independent findings.

**TABLE 1:** Demographic and cognitive characteristics.

Characteristic	NA (a)	SCA (b)	MA (c)
Age (Mean ± SD)	84.63 ± 4.02	85.27 ± 5.60	56.87 ± 4.02
Sex: Female/Male	20/9	20/9	20/9
Education (year)	17.14 ± 2.67	16.21 ± 2.51	17.411.90
RAVLT	5.791.54	10.441.38a*	10.411.55 a*
TMT-A	38.6410.31	35.8611.40c*	24.017.13 a*b*
TMT-B	102.2135.02	78.80 ± 29.72a*c*	52.0416.03a*b*
MoCA	24.972.44	25.622.56c*	27.791.92a*b*

a, b, and c represent the three groups NA, SCA, and MA, respectively. Superscript letters denote a significant difference compared to the specific group designated by that letter. For example, a *indicates a significant difference compared to the NA group at *p <* 0.05. NA, normal aging; SCA, successful cognitive aging; MA, middle-aged adults; RAVLT, Rey Auditory Verbal Learning Test; TMT-A, Trail Making Test Part A; TMT-B, Trail Making Test Part B; MoCA, Montreal Cognitive Assessment.

### SCA and NA groups share similar macroscopic structure

Across all three PCC subregions (dPCC, vPCC, and RSC) in both hemispheres, no statistically significant differences in cortical thickness were observed between the SCA and NA groups (*p* > 0.05). However, compared to the younger MA group, both older adult groups (SCA and NA) exhibited significantly reduced cortical thickness in the bilateral dPCC and vPCC (*p* < 0.05), whereas the thickness reduction in the bilateral RSC did not reach statistical significance. Supplementary Table 1 presents the detailed results of these comparisons.

### Exploratory FWF difference in the left vPCC

NODDI analysis provided further insights into tissue organization beyond cortical thickness. Across all bilateral PCC subregions (dPCC, vPCC, and RSC), no statistically significant differences were observed between the SCA and NA groups in either the NDI or the ODI (*p* > 0.05). Analysis of FWF showed no significant differences between the SCA and NA groups across the PCC subregions. However, in the left vPCC, FWF was numerically lower in the SCA group than in the NA group (0.124 ± 0.066 vs. 0.164 ± 0.078), but this comparison did not reach significance (Cohen’s d = 0.625, Tukey-adjusted *p* = 0.059) and was therefore considered exploratory. Relative to the MA group (0.073 ± 0.029), both older groups showed significantly higher left vPCC FWF (both adjusted *p <* 0.01). In the remaining PCC subregions, no other significant group differences or trends between the SCA and NA groups were found for the FWF. Supplementary Tables 2–4 present the detailed results of these comparisons.

### Widespread reduction of functional connectivity between the tripartite PCC and prefrontal cortex in SCA

The functional connectivity (FC) analysis revealed that there were distinct connection patterns and extensive differences in large-scale network organization among the three subregions of the posterior cingulate cortex (PCC) (see Figure 1B). Compared to the NA group, the SCA group exhibited significantly weaker connectivity between all three PCC subregions and extensive prefrontal cortical areas (FDR-adjusted *p <* 0.05). Specifically, this reduced connectivity pattern in the SCA group was observed between the left vPCC and the medial prefrontal cortex (e.g., bilateral 9m and right 10d), between the right dPCC and the anterior cingulate cortex (e.g., right d32 and p32), and between the left RSC and the dorsolateral prefrontal/parahippocampal cortices (e.g., left p9_46v and PHA1) (see Figures 1C, D; for full details, see Supplementary Table 5).

Brain–behavior correlations were examined in the combined older-adult sample (SCA and NA; *n* = 58; Figure 1E). Lower left vPCC–left area 9m connectivity was associated with higher RAVLT delayed-recall scores (*r* = −0.430, raw *P* = 0.0012, FDR-adjusted *P* = 0.026). However, this association was not significant within either the SCA group (*r* = −0.085, *P* = 0.687, FDR-adjusted *P* = 0.890) or the NA group (*r* = −0.164, *P* = 0.433, FDR-adjusted *P* = 0.714). Because the pooled association may reflect between-group differences, it was considered exploratory despite surviving FDR correction. Three additional associations reached nominal significance but did not survive FDR correction: left vPCC–right area 9m connectivity with TMT-B performance (*r* = 0.320, *P* = 0.018), left dPCC–left area 6a connectivity with RAVLT performance (*r* = −0.346, *P* = 0.010), and left RSC–left PHA1 connectivity with RAVLT performance (*r* = −0.315, *P* = 0.020). These associations were also considered exploratory. Complete correlation results are provided in Supplementary Table 6.

### Sensitivity analysis of site-related variability

Acquisition-site distributions did not differ significantly among the MA, NA, and SCA groups (Fisher’s exact *P* = 0.14). In the ComBat-based sensitivity analysis, the principal conclusions for cortical thickness, NDI, and ODI remained unchanged, while the numerical left vPCC FWF difference between the SCA and NA groups remained nonsignificant and exploratory (Tukey-adjusted *P* = 0.062, Cohen’s d = 0.619). All 11 functional-connectivity edges identified in the primary analysis retained the same direction of group difference after harmonization. Six retained an FDR-significant omnibus group effect, and four also retained a significant Tukey-adjusted SCA–NA comparison. Thus, the overall direction and principal spatial pattern were broadly consistent with the primary findings, although the edge-level statistical evidence was attenuated. Complete results are provided in Supplementary Tables 7–9.

## Discussion

In the current study, we investigated the structural and functional characteristics of PCC subregions in individuals with SCA using a multimodal magnetic resonance imaging approach (structural MRI, NODDI, and rs-fMRI). Our findings demonstrate that SCA was not associated with a significant preservation of cortical thickness relative to normal aging. Instead, SCA was primarily associated with weaker resting-state functional connectivity between all PCC subregions and widespread prefrontal cortices. FWF was also numerically lower in the left vPCC in SCA than in NA, but this difference did not reach significance after Tukey adjustment and was considered exploratory. These results support the functional and structural heterogeneity of PCC subregions during aging. Furthermore, they suggest that SCA may be related to broader alterations in PCC–prefrontal functional synchronization, rather than uniform structural preservation of the PCC. Although exploratory, the numerical FWF difference tentatively highlights the left vPCC as a candidate region for future microstructural investigation and independent validation.

Because RAVLT and TMT-B formed part of the group definitions, differences in these measures were expected. MoCA scores, however, did not differ significantly between the SCA and NA groups, suggesting that the group distinction was not evident on a global cognitive screening measure. Considered alongside the imaging findings, this pattern is consistent with the possibility that SCA is characterized by domain- and network-specific features rather than uniformly superior global cognition or widespread structural preservation. Nevertheless, the absence of a significant MoCA difference should not be interpreted as evidence of equivalent global cognition.

Regarding cortical morphology, we observed no significant differences in cortical thickness between the SCA and NA groups across the three PCC subregions. This finding differs from some previous reports of structural preservation in SCA, particularly in the ACC, hippocampus, and prefrontal cortex, although PCC preservation has also been identified in several whole-brain analyses (Harrison et al., 2012; Pezzoli et al., 2024; Xu et al., 2023). Despite enduring this age-related atrophy, the SCA group maintained intact higher-order cognitive functions. This phenomenon aligns with the classic “cognitive reserve” hypothesis (Stern et al., 2020), which posits that the adaptability of cognitive processes (including efficiency, capacity, and flexibility) enables individuals to actively compensate and maintain optimal cognitive and daily functioning when facing structural brain aging or pathological damage. At the neuromechanistic level, this reserve capacity is generally considered to be supported by highly adaptive brain functional network processes. This implies that the SCA cohort’s advantage in resisting cognitive decline may not solely rely on the preservation of macroscopic anatomy; instead, the neural basis of their cognitive advantage likely resides in finer cortical organizational properties or functional connectivity profiles.

In the NODDI-based analysis of cortical microstructural properties, neither NDI nor ODI differed significantly between the SCA and NA groups. FWF was numerically lower in the left vPCC in SCA than in NA, but this exploratory comparison did not reach significance after Tukey adjustment (*P* = 0.0589). In the NODDI model, FWF estimates the relative contribution of an isotropically diffusing, free-water-like compartment rather than directly measuring extracellular space or tissue integrity (Pasternak et al., 2009). During normal aging, cortical atrophy or decreased dendritic spine density often leads to the expansion of the extracellular space, subsequently causing elevated FWF (Lee et al., 2024; Rathi et al., 2014). Accordingly, the numerical FWF difference in the left vPCC may indicate variation in local water-compartment properties, but its underlying biological basis cannot be determined from the present data. This hypothesis-generating observation suggests that left vPCC FWF may provide information complementary to cortical thickness, but it does not establish a microstructural basis for preserved cognition and requires independent replication.

At the resting-state functional connectivity level, fine-grained subregional analysis showed that the SCA group had weaker connectivity than the NA group for selected edges involving all three PCC subregions and predominantly prefrontal regions. This finding differs from some previous neuroimaging studies emphasizing enhanced network connectivity in successful cognitive aging (Raichlen and Alexander, 2014; Zhang et al., 2020). However, previous findings do not indicate that connectivity is uniformly enhanced in successful aging. Lin et al. reported both stronger and weaker cingulate connectivity in older adults with excellent memory (Lin et al., 2017). Thus, successful cognitive aging may involve connection- and subregion-specific functional reorganization rather than a unidirectional increase in connectivity. Differences in spatial analytical scale may partly account for the heterogeneous findings across studies. Analyses treating the DMN or the PCC as a homogeneous entity may average across divergent subregional effects. By separately examining the dPCC, vPCC, and RSC, the present study may have been more sensitive to localized connectivity differences between specific PCC subregions and prefrontal cortical areas. Nevertheless, differences in SCA definitions, sample characteristics, and analytical procedures may also contribute to the discrepancies across studies.

Traditional accounts propose that elevated resting-state connectivity in older adults may compensate for age-related structural deterioration (Cabeza et al., 2018). However, stronger connectivity is not necessarily associated with better cognitive function (Hillary et al., 2015). Chan et al. found that increasing age was accompanied by reduced segregation of large-scale brain systems and that greater segregation of association systems predicted better long-term memory independently of age (Chan et al., 2014). Similarly, Nashiro et al. reported that older adults exhibited increased connectivity outside several cognitive networks, consistent with network dedifferentiation, and that both reduced within-network connectivity and increased out-of-network connectivity were associated with poorer cognitive performance (Nashiro et al., 2017). These findings suggest that excessive or nonspecific cross-regional synchronization may sometimes reflect reduced functional specialization rather than effective compensation. Consistent with this possibility, stronger connectivity between the left vPCC and medial prefrontal cortex (left area 9m) was associated with poorer episodic memory in the combined older-adult sample. However, this association was not significant within either the SCA or NA group. Moreover, because RAVLT performance contributed to the operational group definition, the pooled association may partly reflect the expected cognitive separation between the groups. The inverse association is therefore compatible with, but does not establish, the possibility that weaker PCC–prefrontal coupling reflects greater neural efficiency or reduced network dedifferentiation. Stronger connectivity in NA cannot be considered definitively maladaptive, nor can weaker connectivity in SCA be assumed to be protective.

Further caution is warranted because both the direction of functional connectivity changes and their associations with cognitive performance may vary across stages of aging and pathology. Schultz et al. demonstrated a nonlinear relationship between Alzheimer-related pathology and connectivity in clinically normal older adults (Schultz et al., 2017). Amyloid-positive individuals showed increased DMN and salience-network connectivity when neocortical tau levels were low, followed by reduced connectivity as tau burden increased. Complementing this pathology-related pattern, Staffaroni et al. reported a nonlinear longitudinal trajectory of within-DMN connectivity in cognitively normal adults aged 49–87 years (Staffaroni et al., 2018). Connectivity tended to increase between approximately 50 and 66 years of age but declined at an accelerating rate after age 74. Longitudinal changes in within-DMN connectivity were associated with changes in episodic memory and processing speed, although the latter association was attenuated after additional adjustment. These findings indicate that neither hyperconnectivity nor hypoconnectivity should be interpreted as uniformly adaptive or pathological. In the present study, the direction and principal spatial distribution of the FC findings remained broadly consistent after ComBat harmonization, although fewer individual edges met the full significance criteria. This attenuation supports interpreting the overall connectivity pattern more cautiously than any single edge. Longitudinal studies incorporating molecular biomarkers and direct measures of network segregation are needed to distinguish neural efficiency from preserved network differentiation, emerging disconnection, or other age-related processes.

Several limitations should be considered. First, the cross-sectional design precludes causal inference and cannot distinguish stable characteristics of SCA from longitudinal changes. Second, the modest sample size (*n* = 29 per group) limited statistical power and estimation precision, particularly for subtle microstructural differences across PCC subregions. Therefore, the nonsignificant findings for cortical thickness, NDI, and ODI do not establish group equivalence. The left vPCC FWF difference also remained nonsignificant after Tukey adjustment and should be considered exploratory. Third, SCA was defined using selected measures of episodic memory and executive function rather than a comprehensive multidomain or longitudinal assessment. Finally, residual confounding by unmeasured genetic, vascular/metabolic, and medication-related factors cannot be excluded. Although education was matched across groups, the highly educated volunteer sample may limit generalizability to more diverse populations. Larger, more diverse longitudinal cohorts are needed to evaluate the robustness, temporal stability, and clinical relevance of these findings.

In summary, this multimodal study characterizes subregion-specific PCC features associated with SCA. In this sample, SCA was not accompanied by detectable preservation of PCC cortical thickness but was associated with weaker resting-state connectivity across selected PCC–prefrontal edges. The left vPCC showed an exploratory numerical FWF difference and altered medial prefrontal connectivity, with weaker connectivity associated with better episodic memory in the combined older-adult sample. These findings identify the left vPCC as a candidate for further investigation and are consistent with an inverse association between specific resting-state connections and cognition, but they do not establish a pivotal, protective, or causal role. Larger longitudinal cohorts are needed to characterize the trajectories of these measures and evaluate their potential as neuroimaging biomarkers of cognitive aging.

## Data Availability

The original contributions presented in the study are included in the article/Supplementary material, further inquiries can be directed to the corresponding author.

## Footnotes

1
https://balsa.wustl.edu/file/show/3VLx
