# Correction: Structural and functional alterations of posterior cingulate cortex subregions in successful cognitive aging

**DOI:** 10.3389/fnagi.2026.1997642

**Published:** 2026-10-14

**Authors:** Jie Wang, Yunfei Li, Tianyuyi Feng, Ruixia Xu, Jiaqi Li, Yanling Zheng, Yang Hu, Xiaohu Zhao

**Affiliations:** 1 The Department of Radiology of the Fifth People's Hospital of Shanghai, Fudan University, Shanghai, China; 2 Department of Radiology, Sichuan Provincial People's Hospital, University of Electronic Science and Technology of China, Chengdu, China; 3 Department of Radiology, Deyang People's Hospital, Deyang, China; 4 Chongqing BLCX Technology Co., Ltd., Chongqing, China

**Keywords:** cortical microstructure, functional connectivity, multimodal neuroimaging, posterior cingulate cortex, successful cognitive aging

In the published version of the article, the images for Figures 1, 2 were inadvertently interchanged. The image intended for Figure 1, showing the posterior cingulate cortex subregions, functional connectivity alterations, and their association with episodic memory, was displayed as Figure 2. Conversely, the image intended for Figure 2, showing the participant grouping and multimodal neuroimaging analysis workflow, was displayed as Figure 1.

**Figure 1:**
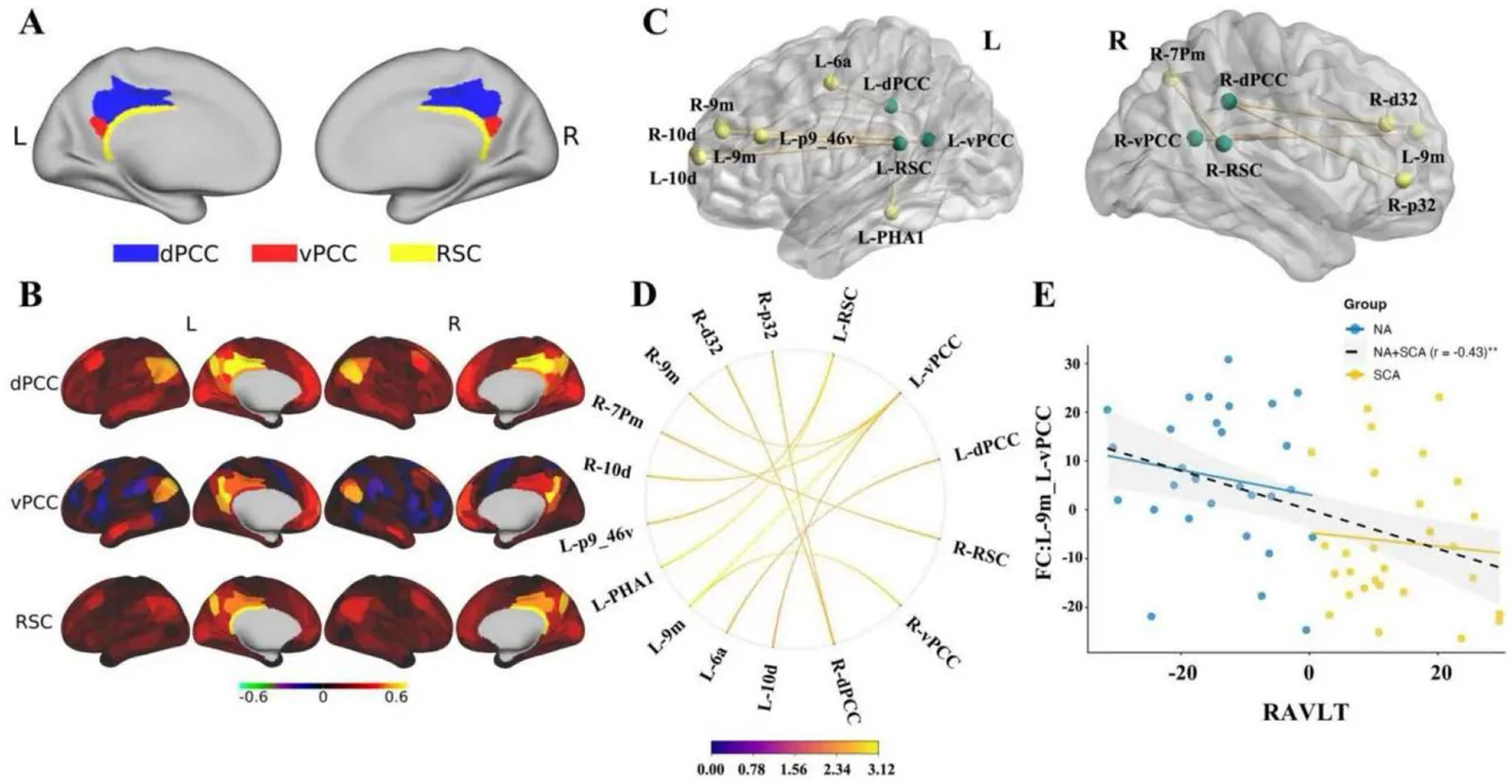
Subregion-specific functional connectivity alterations in successful cognitive aging and their associations with episodic memory. **(A)** Parcellation of the posterior cingulate cortex (PCC) based on the HCP-MMP atlas into the dorsal PCC (dPCC, blue), ventral PCC (vPCC, red), and retrosplenial cortex (RSC, yellow). **(B)** Seed-based resting-state functional connectivity (FC) profiles of the dPCC, vPCC, and RSC across both hemispheres. The color scale represents Pearson's correlation coefficients. **(C)** Three-dimensional cortical visualization of FC edges showing lower connectivity in the SCA group than in the NA group. The labels L and R denote edge sets involving left- and right-hemisphere PCC seed nodes, respectively, rather than the hemispheric location of every connected target region. Target regions may therefore be located in either hemisphere. The identified edges predominantly connected PCC subregions with prefrontal regions, including the medial prefrontal cortex (e.g., areas 9m and 10d) and anterior cingulate cortex (e.g., areas d32 and p32). **(D)** Circular chord diagram summarizing the region-to-region FC differences between the SCA and NA groups. Edge colors represent the T statistics for the NA > SCA contrast. Edges shown in panels C and D satisfied an FDR-adjusted omnibus group effect followed by a significant SCA–NA post hoc comparison (Tukey-adjusted *P* < 0.05). **(E)** Association between left vPCC–left area 9m connectivity and RAVLT delayed-recall performance. Partial Spearman correlation analysis, adjusted for age, sex, years of education, and mean framewise displacement, showed a significant negative association in the combined older-adult sample (SCA and NA, *n* = 58; *r* = −0.430, raw *P* = 0.0012, FDR-adjusted *P* = 0.026). The association was not significant within the SCA group (*n* = 29; *r* = −0.085, *P* = 0.687) or the NA group (*n* = 29; *r* = −0.164, *P* = 0.433). The black dashed line represents the pooled fit, whereas the colored solid lines represent group-specific fits. The gray band denotes the 95% confidence interval for the pooled fit. Because the within-group associations were not significant, the pooled association may partly reflect between-group differences and should be interpreted accordingly.

**Figure 2:**
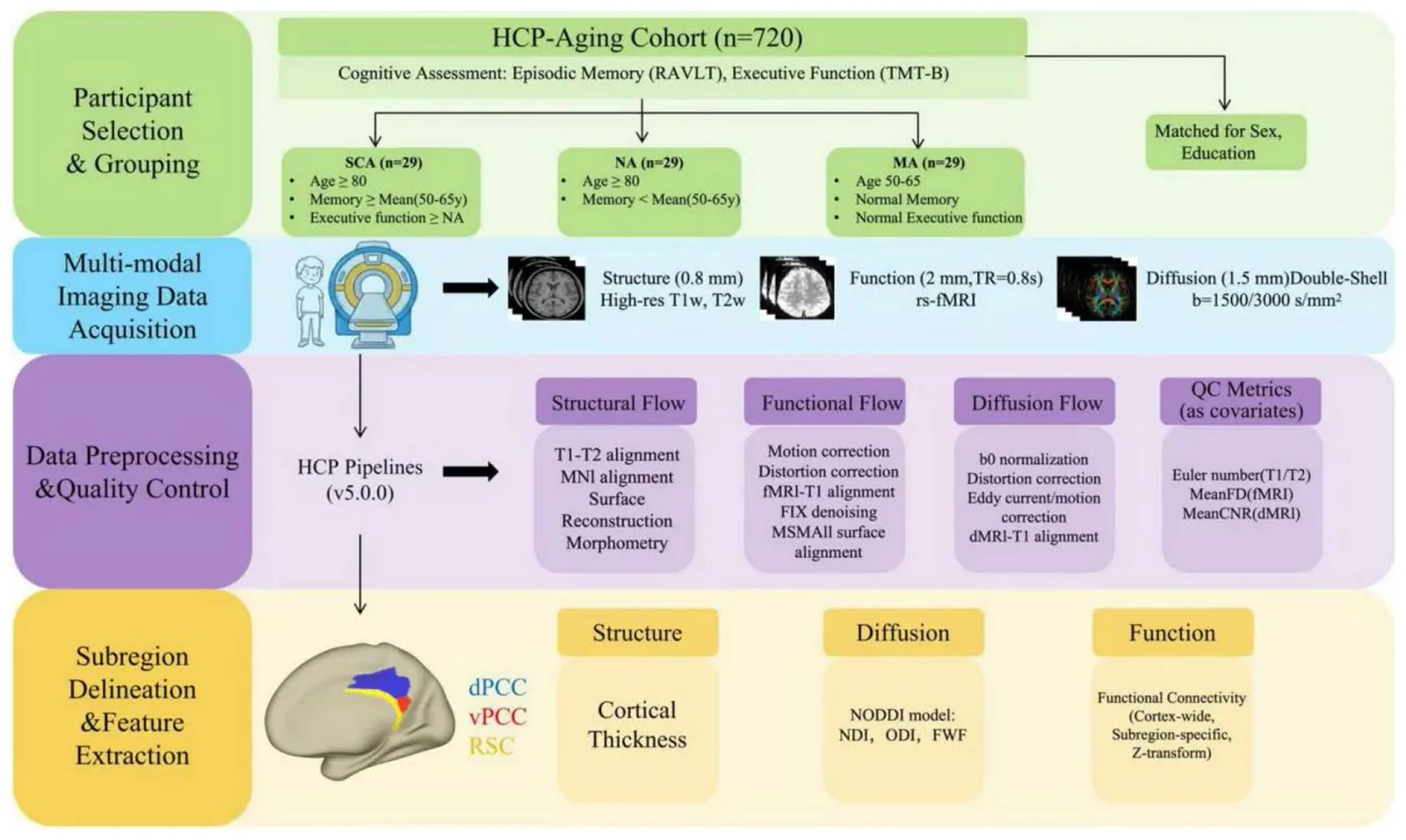
Flowchart of participant grouping and multimodal neuroimaging analysis based on the HCP-Aging cohort. The analytical pipeline comprised four main steps. (1) Participant grouping: Participants were classified as successful cognitive aging (SCA), normal aging (NA), or middle-aged adults (MA) based on RAVLT and TMT-B performance. NA and MA participants were matched to the SCA group for sex and years of education, yielding 29 participants per group. (2) Data acquisition: High-resolution T1- and T2-weighted structural MRI, resting-state functional MRI (rs-fMRI), and double-shell diffusion MRI (dMRI) data were acquired. (3) Preprocessing and quality control: Multimodal MRI data were processed using the HCP Pipelines (v5.0.0). Euler number, mean framewise displacement (MeanFD), and mean contrast-to-noise ratio (MeanCNR) were used as modality-specific image-quality covariates. (4) Feature extraction: Cortical thickness, NODDI-derived microstructural parameters (NDI, ODI, and FWF), and cortex-wide functional connectivity were extracted for the dPCC, vPCC, and RSC. SCA, successful cognitive aging; NA, normal aging; MA, middle-aged adults; RAVLT, Rey Auditory Verbal Learning Test; TMT-B, Trail Making Test Part B; PCC, posterior cingulate cortex; dPCC, dorsal posterior cingulate cortex; vPCC, ventral posterior cingulate cortex; RSC, retrosplenial cortex; NODDI, neurite orientation dispersion and density imaging; NDI, neurite density index; ODI, orientation dispersion index; FWF, free water fraction; MeanFD, mean framewise displacement; MeanCNR, mean contrast-to-noise ratio.

The figure numbers and figure captions were presented in the correct order and remain unchanged. Only the two figure images have been exchanged. This correction does not affect the data, results, interpretations, or conclusions of the article. The corrected Figures 1, 2, along with their captions, appear below.

The original version of this article has been updated.

