# Supplementary material for: Structural and functional alterations of posterior cingulate cortex subregions in successful cognitive aging
Source: Front Aging Neurosci. 2026 Sep 1;18:1929453. doi: 10.3389/fnagi.2026.1929453 (PMC13574787; doi:10.3389/fnagi.2026.1929453)
Supplement: Supplementary file 1 [file Data_Sheet_1.docx]

**Supplementary_Material**

### **Supplementary Methods**

### **Supplementary Table 1-9**

### **Supplementary Figure 1**

### Supplementary Methods

#### MRI acquisition parameters

MRI data were acquired at four HCP-Aging sites using Siemens 3T Prisma scanners equipped with 80 mT/m gradients and 32-channel head coils. All scanners used the same E11C software version and an electronically distributed acquisition protocol. Standardized operating procedures, cross-site training, and centralized quality-control procedures were implemented to promote consistency across sites. Only the imaging modalities used in the present study are described below.

#### Structural MRI

Each participant underwent one T1-weighted and one T2-weighted acquisition at 0.8-mm isotropic resolution. T1-weighted images were acquired using a sagittal four-echo magnetization-prepared rapid gradient-echo sequence with embedded volumetric navigators for prospective motion correction and selective reacquisition. The acquisition parameters were as follows: repetition time (TR) = 2,500 ms; echo times (TEs) = 1.81, 3.60, 5.39, and 7.18 ms; inversion time (TI) = 1,000 ms; flip angle = 8°; field of view = approximately 256 × 240 × 166 mm³; acquisition matrix = 320 × 300 × 208; generalized autocalibrating partially parallel acquisition factor = 2; and 6/8 slice partial Fourier. The four echoes were combined to generate the final T1-weighted image.

T2-weighted images were acquired using a sagittal variable-flip-angle turbo spin-echo sequence (Siemens SPACE), also incorporating volumetric navigators. The acquisition parameters were as follows: TR = 3,200 ms; TE = 564 ms; field of view = approximately 256 × 240 × 166 mm³; acquisition matrix = 320 × 300 × 208; 208 slices; and generalized autocalibrating partially parallel acquisition factor = 2.

#### Resting-state functional MRI

Resting-state functional MRI data were acquired using a multiband gradient-echo echo-planar imaging sequence. Four runs were collected across two imaging sessions, comprising two runs with anterior-to-posterior and two with posterior-to-anterior phase encoding. Each run contained 488 volumes acquired at 2-mm isotropic resolution with the following parameters: TR = 800 ms; TE = 37 ms; flip angle = 52°; multiband acceleration factor = 8; field of view = 208 × 208 mm²; acquisition matrix = 104 × 104; 72 slices; and receiver bandwidth = 2,290 Hz/pixel. Each run had a nominal time-series duration of approximately 6.5 min. Participants were instructed to remain still and maintain fixation. Spin-echo images with reversed anterior-to-posterior and posterior-to-anterior phase-encoding directions were acquired for susceptibility-induced distortion correction.

#### Diffusion MRI

Diffusion MRI data were acquired using a multiband spin-echo echo-planar imaging sequence at 1.5-mm isotropic resolution. The acquisition parameters were as follows: TR = 3,230 ms; TE = 89.2 ms; excitation flip angle = 78°; refocusing flip angle = 160°; multiband acceleration factor = 4; field of view = 210 × 210 mm²; acquisition matrix = 140 × 140; 92 slices; receiver bandwidth = 1,700 Hz/pixel; and 6/8 phase partial Fourier.

Four diffusion runs were acquired using complementary 98- and 99-entry gradient tables, with each table collected once using anterior-to-posterior and once using posterior-to-anterior phase encoding. Each gradient table contained seven b=0 entries, yielding 91 and 92 diffusion-weighted volumes in the 98- and 99-entry runs, respectively. The nonzero diffusion-weighted volumes were distributed across two interleaved shells with b-values of 1,500 and 3,000 s/mm². Across the complementary gradient tables, 92–93 unique diffusion directions were sampled per shell. The nominal acquisition durations were approximately 5 min 38 s for each 98-entry run and 5 min 42 s for each 99-entry run.

Supplementary Table 1. Group differences in PCC cortical thickness

**A. Omnibus group effects**

| **Subregion** | **MA** | **NA** | **SCA** | **F** | **Raw P** | **FDR P** |
| --- | --- | --- | --- | --- | --- | --- |
| R_RSC | 2.039±0.266 | 1.931±0.274 | 1.958±0.289 | 0.920 | 0.403 | 0.403 |
| R_dPCC | 2.62±0.192 | 2.353±0.178 | 2.342±0.19 | 14.754 | 3.44e-06 | 1.03e-05 |
| R_vPCC | 2.948±0.262 | 2.682±0.25 | 2.693±0.253 | 5.735 | 0.0047 | 0.0084 |
| L_RSC | 2.151±0.3 | 1.944±0.228 | 1.963±0.336 | 1.463 | 0.238 | 0.285 |
| L_dPCC | 2.673±0.172 | 2.349±0.181 | 2.367±0.173 | 16.209 | 1.20e-06 | 7.20e-06 |
| L_vPCC | 3±0.19 | 2.749±0.282 | 2.724±0.284 | 5.532 | 0.0056 | 0.0084 |

**B. Tukey-adjusted pairwise comparisons**

| **Subregion** | **MA–NA Tukey P** | **MA–SCA Tukey P** | **NA–SCA Tukey P** | **Cohen's d (NA–SCA)** |
| --- | --- | --- | --- | --- |
| R_RSC | 0.372 | 0.611 | 0.869 | -0.135 |
| R_dPCC | 1.93e-05 | 1.13e-05 | 1.000 | -0.007 |
| R_vPCC | 0.0079 | 0.0093 | 0.983 | -0.048 |
| L_RSC | 0.234 | 0.347 | 0.938 | -0.091 |
| L_dPCC | 4.76e-06 | 7.02e-06 | 0.950 | -0.081 |
| L_vPCC | 0.0280 | 0.0049 | 0.820 | 0.161 |

Note: Group values are unadjusted mean ± standard deviation. Omnibus group effects were tested using ANCOVA with sex, years of education, and Euler number as covariates. Raw P denotes the uncorrected omnibus P value; FDR P was obtained by Benjamini–Hochberg correction across the six bilateral PCC subregions for the corresponding metric. Pairwise P values were adjusted using Tukey's procedure. Cohen's d is the model-based standardized NA–SCA contrast; positive values indicate higher values in NA. MA, middle-aged adults; NA, normal aging; SCA, successful cognitive aging; L, left; R, right; dPCC, dorsal posterior cingulate cortex; vPCC, ventral posterior cingulate cortex; RSC, retrosplenial cortex.

Supplementary Table 2. Group differences in PCC neurite density index (NDI)

**A. Omnibus group effects**

| **Subregion** | **MA** | **NA** | **SCA** | **F** | **Raw P** | **FDR P** |
| --- | --- | --- | --- | --- | --- | --- |
| R_RSC | 0.294±0.017 | 0.31±0.021 | 0.311±0.027 | 4.072 | 0.0206 | 0.0413 |
| R_dPCC | 0.245±0.008 | 0.26±0.021 | 0.257±0.017 | 5.306 | 0.0068 | 0.0410 |
| R_vPCC | 0.259±0.013 | 0.276±0.026 | 0.279±0.035 | 2.877 | 0.0621 | 0.0745 |
| L_RSC | 0.297±0.018 | 0.319±0.036 | 0.318±0.037 | 4.255 | 0.0175 | 0.0413 |
| L_dPCC | 0.247±0.009 | 0.256±0.018 | 0.26±0.021 | 2.479 | 0.0902 | 0.0902 |
| L_vPCC | 0.256±0.012 | 0.275±0.031 | 0.271±0.029 | 3.309 | 0.0416 | 0.0624 |

**B. Tukey-adjusted pairwise comparisons**

| **Subregion** | **MA–NA Tukey P** | **MA–SCA Tukey P** | **NA–SCA Tukey P** | **Cohen's d (NA–SCA)** |
| --- | --- | --- | --- | --- |
| R_RSC | 0.0358 | 0.0400 | 0.991 | -0.035 |
| R_dPCC | 0.0050 | 0.0977 | 0.583 | 0.268 |
| R_vPCC | 0.0988 | 0.0924 | 0.982 | -0.048 |
| L_RSC | 0.0298 | 0.0365 | 0.995 | -0.026 |
| L_dPCC | 0.264 | 0.0816 | 0.729 | -0.205 |
| L_vPCC | 0.0355 | 0.189 | 0.796 | 0.174 |

Note: Group values are unadjusted mean ± standard deviation. Omnibus group effects were tested using ANCOVA with sex, years of education, and mean contrast-to-noise ratio as covariates. Raw P denotes the uncorrected omnibus P value; FDR P was obtained by Benjamini–Hochberg correction across the six bilateral PCC subregions for the corresponding metric. Pairwise P values were adjusted using Tukey's procedure. Cohen's d is the model-based standardized NA–SCA contrast; positive values indicate higher values in NA. MA, middle-aged adults; NA, normal aging; SCA, successful cognitive aging; L, left; R, right; dPCC, dorsal posterior cingulate cortex; vPCC, ventral posterior cingulate cortex; RSC, retrosplenial cortex.

Supplementary Table 3. Group differences in PCC orientation dispersion index (ODI)

**A. Omnibus group effects**

| **Subregion** | **MA** | **NA** | **SCA** | **F** | **Raw P** | **FDR P** |
| --- | --- | --- | --- | --- | --- | --- |
| R_RSC | 0.351±0.029 | 0.331±0.055 | 0.339±0.047 | 4.213 | 0.0182 | 0.0363 |
| R_dPCC | 0.364±0.017 | 0.341±0.027 | 0.35±0.022 | 15.545 | 1.93e-06 | 1.16e-05 |
| R_vPCC | 0.341±0.028 | 0.342±0.038 | 0.348±0.032 | 0.131 | 0.877 | 0.877 |
| L_RSC | 0.371±0.033 | 0.361±0.043 | 0.345±0.051 | 2.804 | 0.0665 | 0.0997 |
| L_dPCC | 0.364±0.019 | 0.354±0.027 | 0.347±0.023 | 6.342 | 0.0028 | 0.0083 |
| L_vPCC | 0.35±0.028 | 0.348±0.047 | 0.339±0.028 | 1.072 | 0.347 | 0.417 |

**B. Tukey-adjusted pairwise comparisons**

| **Subregion** | **MA–NA Tukey P** | **MA–SCA Tukey P** | **NA–SCA Tukey P** | **Cohen's d (NA–SCA)** |
| --- | --- | --- | --- | --- |
| R_RSC | 0.0222 | 0.0564 | 0.976 | -0.056 |
| R_dPCC | 1.52e-06 | 5.90e-04 | 0.400 | -0.350 |
| R_vPCC | 0.868 | 0.976 | 0.957 | -0.077 |
| L_RSC | 0.576 | 0.0549 | 0.291 | 0.408 |
| L_dPCC | 0.0747 | 0.0019 | 0.261 | 0.426 |
| L_vPCC | 0.956 | 0.364 | 0.470 | 0.318 |

Note: Group values are unadjusted mean ± standard deviation. Omnibus group effects were tested using ANCOVA with sex, years of education, and mean contrast-to-noise ratio as covariates. Raw P denotes the uncorrected omnibus P value; FDR P was obtained by Benjamini–Hochberg correction across the six bilateral PCC subregions for the corresponding metric. Pairwise P values were adjusted using Tukey's procedure. Cohen's d is the model-based standardized NA–SCA contrast; positive values indicate higher values in NA. MA, middle-aged adults; NA, normal aging; SCA, successful cognitive aging; L, left; R, right; dPCC, dorsal posterior cingulate cortex; vPCC, ventral posterior cingulate cortex; RSC, retrosplenial cortex.

Supplementary Table 4. Group differences in PCC free water fraction (FWF)

**A. Omnibus group effects**

| **Subregion** | **MA** | **NA** | **SCA** | **F** | **Raw P** | **FDR P** |
| --- | --- | --- | --- | --- | --- | --- |
| R_RSC | 0.104±0.039 | 0.166±0.077 | 0.171±0.083 | 5.763 | 0.0046 | 0.0055 |
| R_dPCC | 0.053±0.017 | 0.105±0.055 | 0.115±0.066 | 8.669 | 3.88e-04 | 6.43e-04 |
| R_vPCC | 0.068±0.027 | 0.125±0.09 | 0.143±0.095 | 4.694 | 0.0118 | 0.0118 |
| L_RSC | 0.125±0.05 | 0.219±0.094 | 0.196±0.103 | 8.547 | 4.29e-04 | 6.43e-04 |
| L_dPCC | 0.061±0.018 | 0.12±0.059 | 0.117±0.049 | 12.936 | 1.33e-05 | 4.00e-05 |
| L_vPCC | 0.073±0.029 | 0.164±0.078 | 0.124±0.066 | 15.421 | 2.11e-06 | 1.27e-05 |

**B. Tukey-adjusted pairwise comparisons**

| **Subregion** | **MA–NA Tukey P** | **MA–SCA Tukey P** | **NA–SCA Tukey P** | **Cohen's d (NA–SCA)** |
| --- | --- | --- | --- | --- |
| R_RSC | 0.0104 | 0.0109 | 0.980 | -0.051 |
| R_dPCC | 0.0023 | 8.04e-04 | 0.825 | -0.160 |
| R_vPCC | 0.0494 | 0.0132 | 0.758 | -0.192 |
| L_RSC | 3.54e-04 | 0.0126 | 0.629 | 0.248 |
| L_dPCC | 2.35e-05 | 3.44e-04 | 0.897 | 0.120 |
| L_vPCC | 1.03e-06 | 0.0091 | 0.0590 | 0.625 |

Note: Group values are unadjusted mean ± standard deviation. Omnibus group effects were tested using ANCOVA with sex, years of education, and mean contrast-to-noise ratio as covariates. Raw P denotes the uncorrected omnibus P value; FDR P was obtained by Benjamini–Hochberg correction across the six bilateral PCC subregions for the corresponding metric. Pairwise P values were adjusted using Tukey's procedure. Cohen's d is the model-based standardized NA–SCA contrast; positive values indicate higher values in NA. MA, middle-aged adults; NA, normal aging; SCA, successful cognitive aging; L, left; R, right; dPCC, dorsal posterior cingulate cortex; vPCC, ventral posterior cingulate cortex; RSC, retrosplenial cortex.

Supplementary Table 5. PCC-related functional connectivity edges with weaker connectivity in SCA than NA

**A. Omnibus group effects**

| **Connection** | **MA** | **NA** | **SCA** | **F** | **Raw P** | **FDR P** |
| --- | --- | --- | --- | --- | --- | --- |
| R_7Pm–R_RSC | 0.302±0.112 | 0.271±0.117 | 0.201±0.121 | 6.729 | 0.0020 | 0.0222 |
| R_d32–R_dPCC | 0.428±0.146 | 0.401±0.162 | 0.307±0.111 | 5.808 | 0.0044 | 0.0350 |
| R_p32–R_dPCC | 0.458±0.132 | 0.389±0.164 | 0.29±0.111 | 9.796 | 1.55e-04 | 0.0071 |
| L_9m–R_vPCC | 0.4±0.157 | 0.393±0.121 | 0.301±0.13 | 5.519 | 0.0057 | 0.0396 |
| L_vPCC–R_9m | 0.427±0.164 | 0.405±0.121 | 0.324±0.135 | 6.109 | 0.0034 | 0.0299 |
| L_vPCC–R_10d | 0.513±0.157 | 0.417±0.121 | 0.346±0.126 | 15.982 | 1.41e-06 | 4.19e-04 |
| L_p9_46v–L_RSC | 0.206±0.124 | 0.192±0.151 | 0.104±0.129 | 5.131 | 0.0080 | 0.0448 |
| L_PHA1–L_RSC | 0.127±0.074 | 0.133±0.104 | 0.076±0.063 | 5.359 | 0.0065 | 0.0424 |
| L_6a–L_dPCC | 0.165±0.172 | 0.087±0.123 | 0.001±0.127 | 10.662 | 7.75e-05 | 0.0047 |
| L_9m–L_vPCC | 0.441±0.139 | 0.418±0.129 | 0.325±0.147 | 8.642 | 3.96e-04 | 0.0111 |
| L_10d–L_vPCC | 0.61±0.169 | 0.514±0.12 | 0.444±0.169 | 12.648 | 1.66e-05 | 0.0020 |

**B. Tukey-adjusted pairwise comparisons**

| **Connection** | **MA–NA Tukey P** | **MA–SCA Tukey P** | **NA–SCA Tukey P** | **Cohen's d (NA–SCA)** |
| --- | --- | --- | --- | --- |
| R_7Pm–R_RSC | 0.538 | 0.0017 | 0.0332 | 0.680 |
| R_d32–R_dPCC | 0.704 | 0.0045 | 0.0371 | 0.668 |
| R_p32–R_dPCC | 0.144 | 9.17e-05 | 0.0319 | 0.684 |
| L_9m–R_vPCC | 0.950 | 0.0094 | 0.0202 | 0.731 |
| L_vPCC–R_9m | 0.703 | 0.0035 | 0.0305 | 0.689 |
| L_vPCC–R_10d | 0.0076 | 7.06e-07 | 0.0268 | 0.702 |
| L_p9_46v–L_RSC | 0.891 | 0.0106 | 0.0335 | 0.679 |
| L_PHA1–L_RSC | 0.993 | 0.0185 | 0.0126 | 0.777 |
| L_6a–L_dPCC | 0.0757 | 4.28e-05 | 0.0404 | 0.659 |
| L_9m–L_vPCC | 0.648 | 4.61e-04 | 0.0070 | 0.831 |
| L_10d–L_vPCC | 0.0223 | 8.80e-06 | 0.0493 | 0.637 |

Note: Functional-connectivity values are unadjusted mean ± standard deviation of Fisher Z-transformed correlation coefficients. Omnibus effects were tested using ANCOVA with sex, years of education, and mean framewise displacement as covariates. Raw P denotes the uncorrected omnibus P value, and FDR P was corrected jointly across all 2,079 tested PCC–cortical edges. Pairwise P values were adjusted using Tukey's procedure. Cohen's d is the model-based standardized NA–SCA contrast; positive values indicate higher connectivity in NA. Cortical node labels correspond to the HCP multimodal parcellation. FC, functional connectivity; MA, middle-aged adults; NA, normal aging; SCA, successful cognitive aging.

Supplementary Table 6. Partial correlations between PCC-related functional connectivity and cognitive performance

**A. Combined SCA + NA sample (N = 58)**

| **Measure** | **Connection** | **N** | **Partial r_s** | **Raw P** | **FDR P** |
| --- | --- | --- | --- | --- | --- |
| RAVLT | R_7Pm–R_RSC | 58 | -0.268 | 0.0502 | 0.131 |
| TMT-B | R_7Pm–R_RSC | 58 | 0.009 | 0.949 | 0.949 |
| RAVLT | R_d32–R_dPCC | 58 | -0.205 | 0.137 | 0.201 |
| TMT-B | R_d32–R_dPCC | 58 | 0.175 | 0.206 | 0.283 |
| RAVLT | R_p32–R_dPCC | 58 | -0.164 | 0.235 | 0.304 |
| TMT-B | R_p32–R_dPCC | 58 | 0.210 | 0.127 | 0.200 |
| RAVLT | L_9m–R_vPCC | 58 | -0.357 | 0.0081 | 0.0768 |
| TMT-B | L_9m–R_vPCC | 58 | 0.212 | 0.124 | 0.200 |
| RAVLT | L_vPCC–R_9m | 58 | -0.258 | 0.0597 | 0.131 |
| TMT-B | L_vPCC–R_9m | 58 | 0.320 | 0.0183 | 0.0900 |
| RAVLT | L_vPCC–R_10d | 58 | -0.243 | 0.0764 | 0.153 |
| TMT-B | L_vPCC–R_10d | 58 | 0.263 | 0.0550 | 0.131 |
| RAVLT | L_p9_46v–L_RSC | 58 | -0.262 | 0.0553 | 0.131 |
| TMT-B | L_p9_46v–L_RSC | 58 | -0.042 | 0.761 | 0.797 |
| RAVLT | L_PHA1–L_RSC | 58 | -0.315 | 0.0204 | 0.0900 |
| TMT-B | L_PHA1–L_RSC | 58 | 0.094 | 0.501 | 0.551 |
| RAVLT | L_6a–L_dPCC | 58 | -0.346 | 0.0105 | 0.0768 |
| TMT-B | L_6a–L_dPCC | 58 | -0.156 | 0.259 | 0.317 |
| RAVLT | L_9m–L_vPCC | 58 | -0.430 | 0.0012 | 0.0257 |
| TMT-B | L_9m–L_vPCC | 58 | 0.143 | 0.303 | 0.350 |
| RAVLT | L_10d–L_vPCC | 58 | -0.299 | 0.0283 | 0.104 |
| TMT-B | L_10d–L_vPCC | 58 | 0.220 | 0.110 | 0.200 |

**B. NA group (N = 29)**

| **Measure** | **Connection** | **N** | **Partial r_s** | **Raw P** | **FDR P** |
| --- | --- | --- | --- | --- | --- |
| RAVLT | R_7Pm–R_RSC | 29 | 0.272 | 0.188 | 0.714 |
| TMT-B | R_7Pm–R_RSC | 29 | -0.124 | 0.554 | 0.812 |
| RAVLT | R_d32–R_dPCC | 29 | 0.339 | 0.0975 | 0.714 |
| TMT-B | R_d32–R_dPCC | 29 | 0.179 | 0.392 | 0.714 |
| RAVLT | R_p32–R_dPCC | 29 | 0.366 | 0.0718 | 0.714 |
| TMT-B | R_p32–R_dPCC | 29 | 0.181 | 0.385 | 0.714 |
| RAVLT | L_9m–R_vPCC | 29 | 0.066 | 0.755 | 0.897 |
| TMT-B | L_9m–R_vPCC | 29 | 0.019 | 0.927 | 0.927 |
| RAVLT | L_vPCC–R_9m | 29 | 0.269 | 0.193 | 0.714 |
| TMT-B | L_vPCC–R_9m | 29 | 0.157 | 0.455 | 0.714 |
| RAVLT | L_vPCC–R_10d | 29 | 0.312 | 0.129 | 0.714 |
| TMT-B | L_vPCC–R_10d | 29 | 0.186 | 0.374 | 0.714 |
| RAVLT | L_p9_46v–L_RSC | 29 | 0.027 | 0.899 | 0.927 |
| TMT-B | L_p9_46v–L_RSC | 29 | -0.180 | 0.389 | 0.714 |
| RAVLT | L_PHA1–L_RSC | 29 | -0.094 | 0.655 | 0.847 |
| TMT-B | L_PHA1–L_RSC | 29 | 0.106 | 0.615 | 0.845 |
| RAVLT | L_6a–L_dPCC | 29 | -0.020 | 0.925 | 0.927 |
| TMT-B | L_6a–L_dPCC | 29 | -0.226 | 0.277 | 0.714 |
| RAVLT | L_9m–L_vPCC | 29 | -0.164 | 0.433 | 0.714 |
| TMT-B | L_9m–L_vPCC | 29 | -0.167 | 0.426 | 0.714 |
| RAVLT | L_10d–L_vPCC | 29 | 0.060 | 0.775 | 0.897 |
| TMT-B | L_10d–L_vPCC | 29 | 0.238 | 0.251 | 0.714 |

**C. SCA group (N = 29)**

| **Measure** | **Connection** | **N** | **Partial r_s** | **Raw P** | **FDR P** |
| --- | --- | --- | --- | --- | --- |
| RAVLT | R_7Pm–R_RSC | 29 | -0.281 | 0.173 | 0.763 |
| TMT-B | R_7Pm–R_RSC | 29 | -0.109 | 0.606 | 0.833 |
| RAVLT | R_d32–R_dPCC | 29 | -0.155 | 0.460 | 0.809 |
| TMT-B | R_d32–R_dPCC | 29 | -0.019 | 0.927 | 0.927 |
| RAVLT | R_p32–R_dPCC | 29 | -0.039 | 0.854 | 0.913 |
| TMT-B | R_p32–R_dPCC | 29 | 0.048 | 0.821 | 0.913 |
| RAVLT | L_9m–R_vPCC | 29 | -0.292 | 0.156 | 0.763 |
| TMT-B | L_9m–R_vPCC | 29 | 0.183 | 0.382 | 0.809 |
| RAVLT | L_vPCC–R_9m | 29 | -0.296 | 0.150 | 0.763 |
| TMT-B | L_vPCC–R_9m | 29 | 0.338 | 0.0989 | 0.763 |
| RAVLT | L_vPCC–R_10d | 29 | -0.127 | 0.545 | 0.809 |
| TMT-B | L_vPCC–R_10d | 29 | 0.136 | 0.518 | 0.809 |
| RAVLT | L_p9_46v–L_RSC | 29 | -0.140 | 0.503 | 0.809 |
| TMT-B | L_p9_46v–L_RSC | 29 | -0.125 | 0.552 | 0.809 |
| RAVLT | L_PHA1–L_RSC | 29 | 0.073 | 0.728 | 0.890 |
| TMT-B | L_PHA1–L_RSC | 29 | -0.185 | 0.375 | 0.809 |
| RAVLT | L_6a–L_dPCC | 29 | -0.248 | 0.232 | 0.809 |
| TMT-B | L_6a–L_dPCC | 29 | -0.391 | 0.0535 | 0.763 |
| RAVLT | L_9m–L_vPCC | 29 | -0.085 | 0.687 | 0.890 |
| TMT-B | L_9m–L_vPCC | 29 | 0.159 | 0.446 | 0.809 |
| RAVLT | L_10d–L_vPCC | 29 | -0.136 | 0.517 | 0.809 |
| TMT-B | L_10d–L_vPCC | 29 | 0.034 | 0.871 | 0.913 |

Note: Partial Spearman correlations controlled for age, sex, years of education, and mean framewise displacement. Benjamini–Hochberg correction was applied separately to the 22 tested correlations within each analysis sample. Higher RAVLT delayed-recall scores indicate better episodic memory; longer TMT-B completion times indicate poorer executive function. NA, normal aging; SCA, successful cognitive aging; RAVLT, Rey Auditory Verbal Learning Test delayed recall; TMT-B, Trail Making Test Part B.

Supplementary Table 7. Distribution of acquisition sites across groups

| **Site** | **MA** | **NA** | **SCA** | **Total** |
| --- | --- | --- | --- | --- |
| MGH | 9 (31.0%) | 6 (20.7%) | 6 (20.7%) | 21 |
| UCLA | 5 (17.2%) | 5 (17.2%) | 0 (0.0%) | 10 |
| UMinn | 9 (31.0%) | 10 (34.5%) | 10 (34.5%) | 29 |
| WashU | 6 (20.7%) | 8 (27.6%) | 13 (44.8%) | 27 |
| Total | 29 (100%) | 29 (100%) | 29 (100%) | 87 |

Note: Values are n (percentage within group). Because some site-by-group cell counts were small, group differences in site distribution were evaluated using Fisher's exact test (two-sided P = 0.14). MA, middle-aged adults; NA, normal aging; SCA, successful cognitive aging; MGH, Massachusetts General Hospital; UCLA, University of California, Los Angeles; UMinn, University of Minnesota; WashU, Washington University.

Supplementary Table 8. ComBat-harmonized cortical thickness and NODDI sensitivity analyses

**A. Omnibus group effects**

| **Metric** | **Subregion** | **MA** | **NA** | **SCA** | **F** | **Raw P** | **FDR P** |
| --- | --- | --- | --- | --- | --- | --- | --- |
| Cortical thickness | R_RSC | 2.034±0.253 | 1.929±0.28 | 1.978±0.271 | 1.145 | 0.323 | 0.323 |
| Cortical thickness | R_dPCC | 2.614±0.191 | 2.351±0.17 | 2.35±0.181 | 14.966 | 2.94e-06 | 8.83e-06 |
| Cortical thickness | R_vPCC | 2.941±0.249 | 2.676±0.25 | 2.7±0.232 | 5.871 | 0.0042 | 0.0062 |
| Cortical thickness | L_RSC | 2.149±0.284 | 1.934±0.245 | 1.976±0.308 | 2.233 | 0.114 | 0.137 |
| Cortical thickness | L_dPCC | 2.668±0.166 | 2.348±0.175 | 2.373±0.17 | 16.068 | 1.33e-06 | 7.96e-06 |
| Cortical thickness | L_vPCC | 3.006±0.185 | 2.748±0.276 | 2.718±0.261 | 6.293 | 0.0029 | 0.0058 |
| NDI | R_RSC | 0.295±0.019 | 0.31±0.02 | 0.309±0.025 | 3.727 | 0.0283 | 0.0649 |
| NDI | R_dPCC | 0.246±0.01 | 0.26±0.019 | 0.255±0.015 | 5.669 | 0.0050 | 0.0298 |
| NDI | R_vPCC | 0.261±0.015 | 0.277±0.026 | 0.277±0.033 | 2.597 | 0.0807 | 0.0968 |
| NDI | L_RSC | 0.299±0.023 | 0.317±0.033 | 0.317±0.034 | 3.266 | 0.0433 | 0.0649 |
| NDI | L_dPCC | 0.248±0.011 | 0.256±0.017 | 0.259±0.019 | 1.812 | 0.170 | 0.170 |
| NDI | L_vPCC | 0.257±0.013 | 0.275±0.029 | 0.27±0.027 | 3.430 | 0.0371 | 0.0649 |
| ODI | R_RSC | 0.351±0.029 | 0.331±0.053 | 0.339±0.044 | 4.910 | 0.0097 | 0.0194 |
| ODI | R_dPCC | 0.363±0.017 | 0.341±0.027 | 0.351±0.021 | 17.663 | 4.30e-07 | 2.58e-06 |
| ODI | R_vPCC | 0.34±0.029 | 0.343±0.038 | 0.348±0.03 | 0.061 | 0.941 | 0.941 |
| ODI | L_RSC | 0.372±0.035 | 0.361±0.04 | 0.345±0.048 | 3.302 | 0.0418 | 0.0628 |
| ODI | L_dPCC | 0.363±0.02 | 0.354±0.026 | 0.348±0.021 | 5.817 | 0.0044 | 0.0131 |
| ODI | L_vPCC | 0.348±0.03 | 0.349±0.042 | 0.339±0.026 | 1.086 | 0.343 | 0.411 |
| FWF | R_RSC | 0.108±0.048 | 0.163±0.072 | 0.168±0.078 | 4.647 | 0.0123 | 0.0147 |
| FWF | R_dPCC | 0.057±0.024 | 0.102±0.048 | 0.111±0.063 | 6.521 | 0.0024 | 0.0036 |
| FWF | R_vPCC | 0.077±0.041 | 0.122±0.072 | 0.137±0.089 | 2.882 | 0.0618 | 0.0618 |
| FWF | L_RSC | 0.133±0.064 | 0.217±0.094 | 0.191±0.091 | 7.027 | 0.0015 | 0.0031 |
| FWF | L_dPCC | 0.064±0.022 | 0.12±0.057 | 0.115±0.042 | 12.926 | 1.34e-05 | 4.03e-05 |
| FWF | L_vPCC | 0.077±0.03 | 0.161±0.081 | 0.123±0.054 | 15.121 | 2.63e-06 | 1.58e-05 |

**B. Tukey-adjusted pairwise comparisons**

| **Metric** | **Subregion** | **MA–NA Tukey P** | **MA–SCA Tukey P** | **NA–SCA Tukey P** | **Cohen's d (NA–SCA)** |
| --- | --- | --- | --- | --- | --- |
| Cortical thickness | R_RSC | 0.295 | 0.706 | 0.669 | -0.230 |
| Cortical thickness | R_dPCC | 1.15e-05 | 1.42e-05 | 0.967 | -0.066 |
| Cortical thickness | R_vPCC | 0.0058 | 0.0106 | 0.933 | -0.095 |
| Cortical thickness | L_RSC | 0.0968 | 0.290 | 0.740 | -0.198 |
| Cortical thickness | L_dPCC | 3.80e-06 | 1.10e-05 | 0.872 | -0.134 |
| Cortical thickness | L_vPCC | 0.0227 | 0.0023 | 0.712 | 0.211 |
| NDI | R_RSC | 0.0412 | 0.0598 | 1.000 | -0.002 |
| NDI | R_dPCC | 0.0033 | 0.152 | 0.365 | 0.368 |
| NDI | R_vPCC | 0.0950 | 0.155 | 0.993 | 0.029 |
| NDI | L_RSC | 0.0678 | 0.0733 | 0.992 | -0.033 |
| NDI | L_dPCC | 0.284 | 0.183 | 0.924 | -0.102 |
| NDI | L_vPCC | 0.0286 | 0.263 | 0.623 | 0.251 |
| ODI | R_RSC | 0.0126 | 0.0349 | 0.977 | -0.055 |
| ODI | R_dPCC | 3.54e-07 | 2.14e-04 | 0.377 | -0.362 |
| ODI | R_vPCC | 0.936 | 0.988 | 0.981 | -0.050 |
| ODI | L_RSC | 0.509 | 0.0334 | 0.243 | 0.437 |
| ODI | L_dPCC | 0.0881 | 0.0031 | 0.301 | 0.402 |
| ODI | L_vPCC | 0.998 | 0.450 | 0.369 | 0.366 |
| FWF | R_RSC | 0.0251 | 0.0238 | 0.976 | -0.056 |
| FWF | R_dPCC | 0.0079 | 0.0048 | 0.917 | -0.107 |
| FWF | R_vPCC | 0.143 | 0.0683 | 0.869 | -0.137 |
| FWF | L_RSC | 0.0011 | 0.0457 | 0.503 | 0.303 |
| FWF | L_dPCC | 2.01e-05 | 4.53e-04 | 0.837 | 0.154 |
| FWF | L_vPCC | 1.29e-06 | 0.0099 | 0.0623 | 0.619 |

Note: Imaging measures were harmonized across acquisition sites using ComBat before the original ANCOVA framework was repeated. Group values are mean ± standard deviation after harmonization. FDR P values were corrected across the six bilateral PCC subregions separately for each metric. Pairwise P values were adjusted using Tukey's procedure. Cohen's d is the model-based standardized NA–SCA contrast; positive values indicate higher values in NA. NDI, neurite density index; ODI, orientation dispersion index; FWF, free water fraction.

Supplementary Table 9. ComBat-harmonized functional-connectivity sensitivity analysis

**A. Omnibus group effects**

| **Connection** | **MA** | **NA** | **SCA** | **F** | **Raw P** | **FDR P** |
| --- | --- | --- | --- | --- | --- | --- |
| R_7Pm–R_RSC | 0.298±0.111 | 0.268±0.115 | 0.207±0.12 | 5.648 | 0.0051 | 0.0440 |
| R_d32–R_dPCC | 0.424±0.146 | 0.399±0.148 | 0.314±0.104 | 5.071 | 0.0084 | 0.0574 |
| R_p32–R_dPCC | 0.452±0.135 | 0.389±0.154 | 0.296±0.11 | 8.691 | 3.81e-04 | 0.0122 |
| L_9m–R_vPCC | 0.393±0.153 | 0.391±0.114 | 0.309±0.122 | 4.828 | 0.0104 | 0.0617 |
| L_vPCC–R_9m | 0.417±0.157 | 0.405±0.113 | 0.333±0.137 | 4.954 | 0.0093 | 0.0588 |
| L_vPCC–R_10d | 0.503±0.143 | 0.42±0.118 | 0.351±0.124 | 14.671 | 3.65e-06 | 7.59e-04 |
| L_p9_46v–L_RSC | 0.202±0.118 | 0.181±0.14 | 0.116±0.13 | 3.559 | 0.0330 | 0.121 |
| L_PHA1–L_RSC | 0.125±0.074 | 0.131±0.087 | 0.078±0.066 | 5.139 | 0.0079 | 0.0556 |
| L_6a–L_dPCC | 0.157±0.164 | 0.086±0.119 | 0.008±0.119 | 9.289 | 2.33e-04 | 0.0099 |
| L_9m–L_vPCC | 0.431±0.13 | 0.417±0.129 | 0.334±0.136 | 7.689 | 8.76e-04 | 0.0206 |
| L_10d–L_vPCC | 0.603±0.161 | 0.517±0.114 | 0.45±0.156 | 12.499 | 1.86e-05 | 0.0021 |

**B. Tukey-adjusted pairwise comparisons**

| **Connection** | **MA–NA Tukey P** | **MA–SCA Tukey P** | **NA–SCA Tukey P** | **Cohen's d (NA–SCA)** | **Retained** |
| --- | --- | --- | --- | --- | --- |
| R_7Pm–R_RSC | 0.530 | 0.0041 | 0.0674 | 0.602 | No |
| R_d32–R_dPCC | 0.740 | 0.0085 | 0.0548 | 0.625 | No |
| R_p32–R_dPCC | 0.180 | 2.34e-04 | 0.0456 | 0.646 | Yes |
| L_9m–R_vPCC | 0.982 | 0.0184 | 0.0276 | 0.699 | No |
| L_vPCC–R_9m | 0.841 | 0.0110 | 0.0445 | 0.649 | No |
| L_vPCC–R_10d | 0.0143 | 1.83e-06 | 0.0277 | 0.699 | Yes |
| L_p9_46v–L_RSC | 0.798 | 0.0323 | 0.130 | 0.520 | No |
| L_PHA1–L_RSC | 0.987 | 0.0229 | 0.0141 | 0.766 | No |
| L_6a–L_dPCC | 0.103 | 1.34e-04 | 0.0611 | 0.613 | No |
| L_9m–L_vPCC | 0.782 | 0.0012 | 0.0085 | 0.813 | Yes |
| L_10d–L_vPCC | 0.0258 | 9.81e-06 | 0.0462 | 0.645 | Yes |

Note: The 11 edges identified in the primary analysis are shown after ComBat harmonization. FDR P values were obtained by correction across the complete set of 2,079 ComBat-harmonized PCC–cortical edges. Pairwise P values were adjusted using Tukey's procedure. Retained indicates that both the omnibus FDR-adjusted P value and the NA–SCA Tukey-adjusted P value were <0.05. Cohen's d is the model-based standardized NA–SCA contrast; positive values indicate higher connectivity in NA.

Supplementary Figure 1. Flowchart of participant selection from the HCP-Aging dataset
